# Pseudorabies Virus Variant in Bartha-K61–Vaccinated Pigs, China, 2012

**DOI:** 10.3201/eid1911.130177

**Published:** 2013-11

**Authors:** Tong-Qing An, Jin-Mei Peng, Zhi-Jun Tian, Hong-Yuan Zhao, Na Li, Yi-Min Liu, Jia-Zeng Chen, Chao-Liang Leng, Yan Sun, Dan Chang, Guang-Zhi Tong

**Affiliations:** Chinese Academy of Agricultural Sciences, Harbin, China (T.-Q. An, J.-M. Peng, H.-Y. Zhao, N. Li, Y.-M. Liu, J.-Z. Chen, C.-L. Leng, Y. Sun, D. Chang, Z.-J. Tian, G.-Z. Tong);; Chinese Academy of Agricultural Sciences, Shanghai, China (G.-Z. Tong)

**Keywords:** pseudorabies virus, virus variant, virulent, immune evasion, viruses, pigs, Bartha-K61 vaccine strain, China

## Abstract

The widely used pseudorabies virus (PRV) Bartha-K61 vaccine has played a key role in the eradication of PRV. Since late 2011, however, a disease characterized by neurologic symptoms and a high number of deaths among newborn piglets has occurred among Bartha-K61–vaccinated pigs on many farms in China. Clinical samples from pigs on 15 farms in 6 provinces were examined. The PRV *gE* gene was detectable by PCR in all samples, and sequence analysis of the *gE* gene showed that all isolates belonged to a relatively independent cluster and contained 2 amino acid insertions. A PRV (named HeN1) was isolated and caused transitional fever in pigs. In protection assays, Bartha-K61 vaccine provided 100% protection against lethal challenge with SC (a classical PRV) but only 50% protection against 4 challenges with strain HeN1. The findings suggest that Bartha-K61 vaccine does not provide effective protection against PRV HeN1 infection.

Pseudorabies virus (PRV; family *Herpesviridae*, subfamily *Alphaherpesvirinae*, genus *Varicellovirus*) contains a double-stranded DNA genome with strong genetic stability. The virus has a broad host range and can infect most mammals and some avian species (*1*). Pigs are the natural reservoir for PRV; infection in adult pigs is called Aujeszky disease. Swine farmers with PRV-infected pigs can incur substantial economic costs from reproductive losses in sows and from weight loss in infected adults (*2*). PRV is especially prominent in regions of South America, Asia, and Europe with dense swine populations. There have been no reports of PRV in Norway, Finland, or Malta, and the disease has been eradicated from domestic pig populations in Germany, Austria, Sweden, Denmark, the United Kingdom, Canada, New Zealand, and the United States (*3*). 

The swine industry worldwide has effectively used vaccines to control pseudorabies for >30 years; cases of the disease are now rarely reported from pig farms (*4*–*7*). Among the vaccines, Bartha-K61 is widely used and has played a key role in the eradication of pseudorabies. This vaccine is an attenuated strain of PRV produced by extensive in vitro passage and contains a well-characterized deletion of several viral proteins (i.e., complete gE and US9, partial gI and US2) that attenuates virulence (*8*–*10*). Thus, the gE ELISA is used for the differential diagnosis of infection with field PRV strains or vaccine strain in pigs.

In China, the first report of a PRV outbreak occurred in the 1950s, and the Bartha-K61 vaccine was imported from Hungary to China in the 1970s (*11*). From the 1990s until late 2011, >80% of pigs in China were vaccinated with the Bartha-K61 vaccine, and pseudorabies was well controlled (*12*). However, beginning in late 2011, pseudorabies has occurred on many large pig farms in animals that have been vaccinated with Bartha-K61 vaccine; during the first month of these outbreaks, 50% of samples were positive for pseudorabies gE antibody. The disease is characterized by stillbirth or the birth of weak piglets with neurologic symptoms that ultimately lead to death. The onset of clinical signs in 2- to 3-day-old piglets is sudden, spanning 5 hours from onset to death. The disease in piglets is characterized by shivering and opisthotonos, and 10%–50% of infected piglets die. Since the initial outbreak in late 2011, the disease has occurred in 6 provinces in China with extensive pig-raising industries and caused many piglet deaths and great economic loss (*13*).

The PRV Bartha-K61 vaccine is widely used to protect pigs against pseudorabies; there has been no reported resistance to the vaccine. In 2012, to determine the cause of recent PRV outbreaks among Bartha-K61–vaccinated pigs, we obtained clinical samples from piglets with suspected pseudorabies. A breakthrough PRV was isolated from the samples, and we identified the pathogenicity and immunologic protection of the novel isolate.

## Materials and Methods

In 2012, we collected brain tissue from 154 dead piglets with suspected pseudorabies. The piglets were from 15 farms located in 6 provinces of China: Henan, Heilongjiang, Jilin, Liaoning, Inner Mongolia, and Jiangsu Provinces. All of the farms used PRV Bartha-K61 vaccine to protect their pigs against pseudorabies.

### Cells, Vaccine, and Challenge Virus

Vero cells were used for virus propagation and titration in Dulbecco Modified Eagle Medium (Invitrogen, Carlsbad, CA, USA) supplemented with 10% heat-inactivated fetal bovine serum (Invitrogen), 100 μg/mL streptomycin, and 100 IU/mL penicillin. PRV Bartha-K61 vaccine with a virus titer of 10^5.5^ 50% tissue culture infectious doses (TCID_50_)/dose was purchased from Harbin Weike Biotechnology Development Co. (Harbin, China). According to the quality standards for this vaccine (*14*), the virus titer of qualified product is >5,000 TCID_50_/dose. PRV SC strain, which is highly pathogenic to sheep and pigs (*4*), was isolated in 1980 and has been maintained in our laboratory. This strain has been used as a challenge virus to test the effectiveness of Bartha-K61 vaccine in China from the time the vaccine was first licensed in this country (*14*).

### Viral Genome Extraction and PCR

DNA extraction was performed as described (*15*). The sense primer used for PCR was 5′-ATGCGGCCCTTTCTG-3′, and the reverse primer was 5′-CGGTTCTCCCGGTATTTAAGC-3′. The thermal profile used for PCR was 95°C for 4 min; followed by 35 cycles of 94°C for 30 s, 65°C for 30 s, and 72°C for 2 min; and a final extension at 72°C for 10 min. This generated the complete sequence of the PRV *gE* gene (previously US8) and the flanking regions of *gI* (previously US7), and US9. The PCR products were subjected to electrophoresis on a 1% agarose gel and stained with ethidium bromide for visualization using an ultraviolet transilluminator (MiniLumi; DNR Bio-Imaging Systems Ltd., Kibutz Maale HaHamisha, Israel).

### Virus Isolation

PRV PCR–positive brain tissue homogenates were centrifuged at 10,000 × *g* for 10 min. The supernatant was passed through a 0.45-μm filter and transferred to Vero cell monolayers. The cells were incubated at 37°C and examined daily for cytopathic effect (CPE). After the appearance of CPE, cells were collected and stored at −20°C, and a novel PRV was chosen for further investigation; we named the isolate HeN1. After 3 freeze-thaw cycles, PRV was cultured in Vero cells. The fifth passage of PRV was negatively stained with 2% phosphotungstic acid, and we examined the virus particle morphology by using a transmission electron microscope (H-7650; Hitachi High-Technologies Ltd., Tokyo, Japan).

### Phylogenetic Analyses

Sixteen of the positive PCR products selected from different farms or collection times were cloned into the pMD18-T vector, and the insert was sequenced in both directions. We analyzed sequence data as described (*16*) and compared the complete sequences of the *gE* gene with all PRV *gE* sequences available in the GenBank database (Table 1). We used Lasergene sequence analysis software (DNASTAR, Madison, WI, USA) to perform multiple sequence alignments and phylogenetic analyses.

**Table 1:** Pseudorabies virus isolates whose complete sequences of the gE gene were compared with that of variant HeN1 from pigs vaccinated with Bartha-K61 vaccine strain, China, 2012*

Isolate	Country	Year of isolation	GenBank accession no.
Ea	China	1999	AF171937
PRV-SH	China	1999	AF207700
Guangdong	China	2001	AF403050
LA	China	2002	AY173124
GDSH	China	2007	EF552427
GZ-Z1	China	2010	HQ832846
LXB6	China	2010	GQ926932
LXB88	China	2010	GQ926933
Yangsan	South Korea	2003	AY249861
CL/15	Argentina	1988	JF460026
Kaplan	Hungary	Unknown	JF797218
Becker	United States	Unknown	JF797219
P-PrV	Malaysia	Unknown	FJ176390
Rice	Unknown	1975	M14336
NS374	Belgium	1971	FJ605135
75V19	Belgium	1975	FJ605133
89V87	Belgium	1989	FJ605138
00V72	Belgium	2000	FJ605137
Nia-1	Ireland	1962	FJ605136
NiA3	Spain	2008	EU502923

### Experimental PRV Inoculation of Pigs

Six 3-month-old specific pathogen–free Bama miniature pigs were obtained from the Experimental Animal Center at the Veterinary Research Institute (Harbin, China). All pigs were confirmed to be free of PRV infection by using a gE ELISA kit (HerdChek PRV; IDEXX Laboratories, Westbrook, ME, USA) for PRV antibody detection and by using PCR. The animals were also determined to be free of porcine circovirus type 2, classical swine fever virus, porcine reproductive and respiratory syndrome virus, and swine influenza virus infections by using serologic methods or reverse transcription PCR or PCR as described (*17*,*18*).

The pigs were randomly assigned to 2 rooms and kept under Biosafety Level 2 conditions throughout the experiment. Five of the 6 pigs were in the test group (pigs 011–015); these pigs were injected intramuscularly with a 1-mL inoculum containing 1×10^7.0^ TCID_50_ of PRV strain HeN1. The sixth pig was used as a control and injected intramuscularly with 1 mL of Vero cell culture supernatant. Clinical symptoms were checked daily throughout the study, and rectal temperatures were recorded daily before feeding. We used the HerdChek PRV gE ELISA kit according to the manufacturer’s instructions to analyze PRV-specific antibodies in serum samples collected 0, 2, 5, 7, 14, 21, 28, and 35 days after inoculation. All animals were euthanized on postinoculation day 35. Tissue samples were obtained from the brains, lungs, hearts, testicles, and lymphoid nodes (mandibular, mesenteric, and superficial inguinal) for virus detection by PCR or virus isolation and for histopathologic examination.

### Virus Neutralization Assay

We intramuscularly inoculated 5 PRV-free piglets with 10^5.5^TCID_50_ Bartha-K61 vaccine. Blood samples were collected weekly from each animal, and antisera were individually prepared and stored at −80°C until used. The virus neutralization assay was performed as described (*4*). All sera were heat-inactivated for 30 min at 56°C before testing. The assays were performed by mixing PRV SC and HeN1 strains, respectively, with 50 μL of serially diluted antiserum and 100 TCID_50_ Bartha-K61 vaccine. Antiserum titers were expressed as the highest dilution that reduced the viral CPE by 50% relative to non-neutralized controls. All samples were analyzed in duplicate, and the results shown are the average of the duplicate assays.

### Experimental PRV Inoculation of Sheep

Sheep are commonly used to examine the efficacy of Bartha-K61 vaccine in China. The experimental design, immune dose, and viral challenge level used were in accordance with China’s quality standards for veterinary biological products (*14*). Fourteen 18-month-old sheep were obtained from a farm determined, by PCR and gE ELISA (HerdChek PRV), to be free from PRV infection. Sheep were randomly assigned to 4 isolation rooms. Each sheep in Groups 1 and 3 was injected intramuscularly with 10^5.0^ TCID_50_ Bartha-K61 vaccine. Sheep in Groups 2 and 4 were not vaccinated. After continuous observation for 14 days, the sheep in Groups 1 and 2 were each challenged with 1,000 50% lethal doses (LD_50_) of PRV SC strain, and sheep in Groups 3 and 4 were challenged with 1,000 LD_50_ of HeN1 strain. The LD_50_ was titrated according to quality standards (*14*). According to the quality standards of Bartha-K61 vaccine, at least 2 sheep in the control group would become ill with pseudorabies and die, and all of the vaccinated sheep would be protected against infection.

## Results

### PCR Survey of Clinical Samples

PRV was detected by PCR in 88 (57.1%) of the 154 clinical samples tested. At least 1 sample from each of the 15 farms examined was positive (Table 2), suggesting that wild-type PRV infection is prevalent in China.

**Table 2:** Detection of pseudorabies virus in Bartha-K61–vaccinated pigs on farms in 6 provinces, China, 2012*

Province	No. farms with tested pigs	No. PRV-positive samples/no. tested (%)
Heilongjiang	3	19/26 (73.1)
Jilin	1	2/4 (50.0)
Liaoning	2	11/17 (78.6)
Inner Mongolia	3	17/39 (43.6)
Henan	5	38/67 (53.7)
Jiangsu	1	1/1 (100)
Total	15	88/154 (57.1)

*Virus was detected by gE-specific PCR.

### PRV Isolation

**Figure 1:**
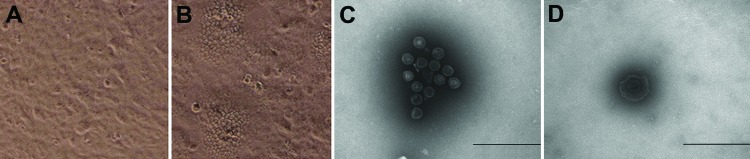
Cytopathic effect and morphology of pseudorabies virus strain HeN1. A) Uninfected control Vero cells. B) Pseudorabies virus–infected Vero cells. A) and B) Original magnification ×200. The cytopathic effect, which was characterized by reticulated cells, was observed 48 h after inoculation. Spherical virus particles without (C) or with (D) viral envelope were observed by electron microscopy. Scale bars indicate 500nm

Positive brain tissue homogenate was filtered to remove bacteria and then inoculated onto Vero cells. CPE was characterized by the appearance of reticulated cells at 48 h (Figure 1). RNA and DNA extracted from the cell cultures were tested by reverse transcription PCR or PCR and were PRV positive but negative for classical swine fever virus and porcine reproductive and respiratory syndrome. The HeN1 isolate was examined by electron microscopy, and spherical viral particles with or without viral envelope were observed (Figure 1).

### Phylogenetic Analysis

**Figure 2:**
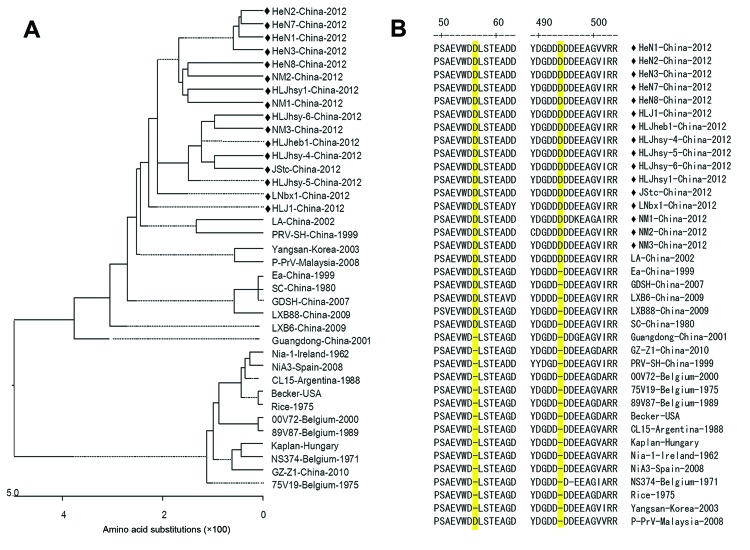
Phylogenetic analysis and comparison, based on gE amino acid sequences, of pseudorabies virus (PRV) isolates. An unrooted tree was constructed from the aligned amino acid sequences of 39 PRV isolates. Black diamonds indicate 16 PRV isolates from China that were collected in 2012; these isolates belong to a relatively independent branch in the phylogenetic tree (A) and possess 2 aspartic acid (Asp, D) insertions (positions 48 and 492–495), which are highlighted in yellow (B).

The complete *gE* genes of 16 isolates collected in 2012 from pig clinical samples were sequenced; each was 1,740 bp. Phylogenetic analysis revealed that the sequences of all 16 isolates clustered to a relatively independent region of the tree; this region was relatively distant from previously isolated strains of PRV (Figure 2, panel A). The PRV isolates shared 98.6%–99.8% aa and 95.0%–99.6% nt identity with previously isolated PRVs. Compared with Kaplan and Becker strains, these 16 isolates contained 2 aa insertions. Aspartate amino acid residues were inserted at positions 48 and 492–495, where the 2012 isolates contained 5 continuous residues and earlier isolates contained 4 continuous residues (Figure 2, panel B). Although amino acid insertion was also observed in a few early Chinese PRV isolates, the insertion in the new 2012 isolates was highly conserved.

### PRV HeN1–Inoculated, Bartha-K61–Vaccinated Pigs

**Figure 3:**
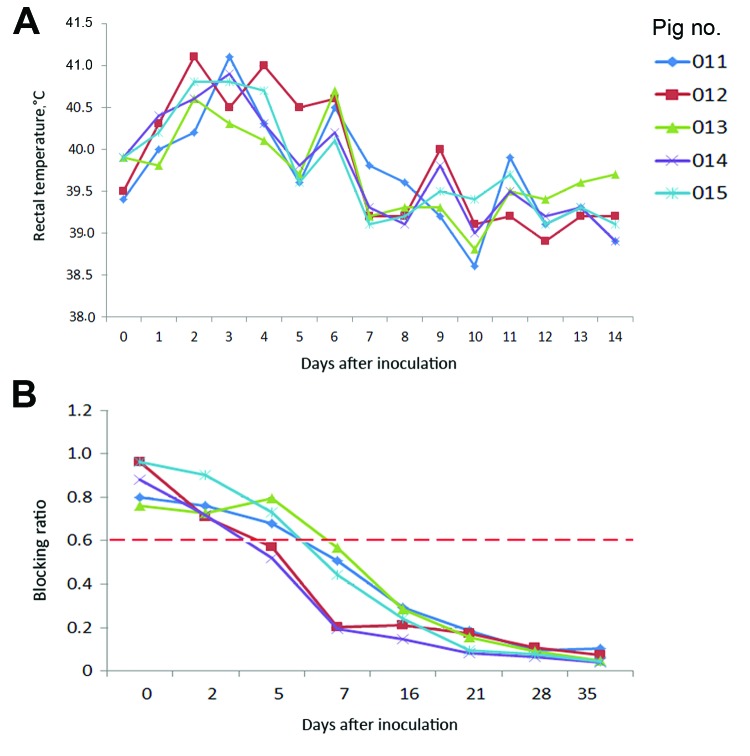
Rectal temperatures and gE antibody levels of Bartha-K61–vaccinated pigs inoculated with pseudorabies virus strain HeN1. A) Rectal temperatures >40.5°C were defined as fever and typically occurred 2–6 days after inoculation. B) Pseudorabies virus gE–specific antibody development was monitored by use of a gE ELISA and reported as blocking ratios; a ratio <0.6 was considered positive.

**Figure 4:**
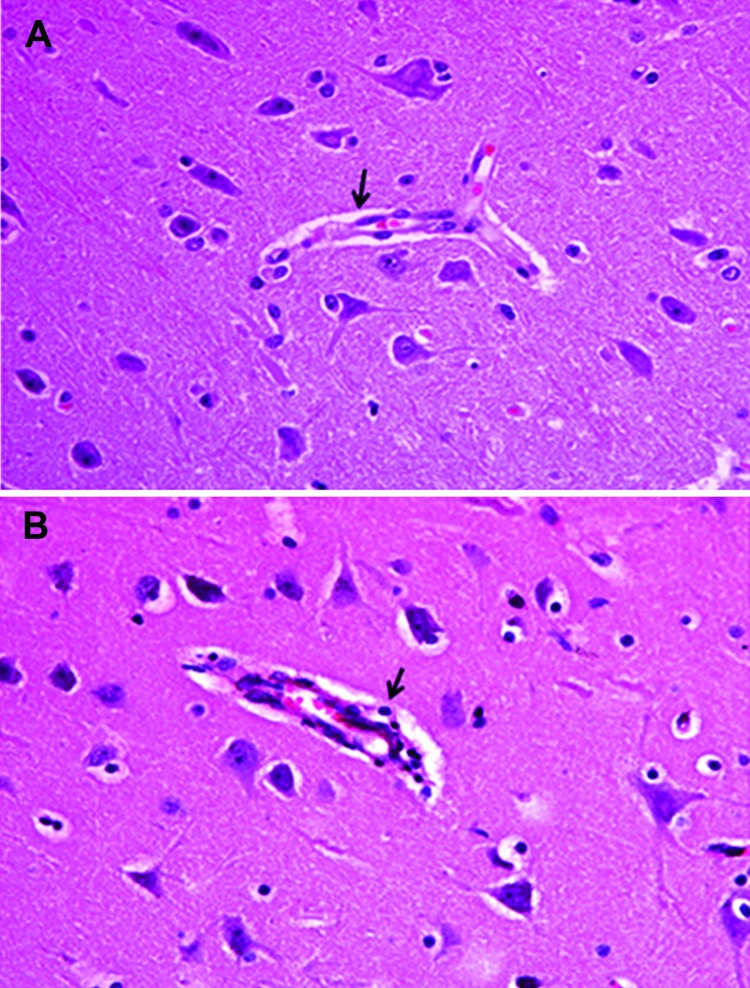
Brain tissue of an unvaccinated control pig (A) and pig inoculated with pseudorabies virus strain HeN1 (B). Arrows indicate lymphocyte infiltration around the small blood vessels in the brain cortex. Original magnification ×100.

Fever (rectal temperature <41.0°C, reference temperature 39.0°C–39.5°C) developed in HeN1 PRV-inoculated pigs 2–6 days after challenge; temperatures returned to normal 7 days after inoculation and remained normal until the end of the experiment (Figure 3). Four to 6 days after challenge, loss of appetite was observed in the pigs; appetites subsequently returned to normal 6–7 days after inoculation without any other clinical symptoms. PRV gE antibodies were detected in serum samples for all pigs 5–7 days after inoculation (Figure 3). Pathologic examination showed brain hemorrhage in all infected pigs (Figure 4); noteworthy damage did not occur in other organs. In the brain, the histopathologic changes were characterized by local bleeding of the meninges, chronic meningitis, and lymphocyte infiltration around the small blood vessels of the brain cortex. PRV HeN1 was not detectable in serum samples by PCR or virus isolation, but it was detectable in all brains and most testicles by PCR (Table 3).

**Table 3:** Virus detection in clinical samples from 5 Bartha-K61–vaccinated pigs inoculated with pseudorabies virus HeN1, China, 2012*

Pig no.	Serum sample on postinoculation day no.			Brain	Lung	Lymph nodes				Testicle		Heart
	2	5	7			Mandibular	Mesenteric		Superficial inguinal			
011	−	−	−	+	+	+	+	−		+	−	
012	−	−	−	+	+	−	+	+		+	−	
013	−	−	−	+	−	−	+	−		+	−	
014	−	−	−	+	−	−	+	−		−	−	
015	−	−	−	+	+	+	−	+		+	−	

*Virus was detected in serum samples by PCR and virus isolation; virus was detected in organs by PCR only.−, negative; +, positive.

### Virus Neutralization Assay

**Figure 5:**
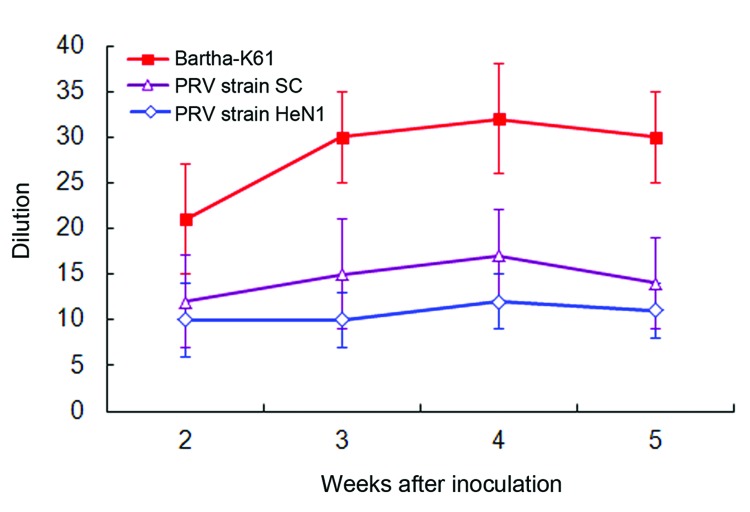
Neutralizing ability of antisera generated against pseudorabies Bartha-K61 vaccine to block wild pseudorabies virus strain infection. The neutralization titer to Bartha-K61 was 20- to 40-fold; the neutralization titers to pseudorabies virus SC and HeN1 strains were 10- to 15-fold and 10-fold, respectively. The virus neutralization assay was performed with antiserum from 5 individual piglets; error bars represent the SD of the 5 experiments.

At different times, we collected antiserum samples from PRV Bartha-K61–vaccinated pigs and measured the virus neutralization capacity against Bartha-K61, SC, and HeN1 PRV strains. The virus neutralization titer of antisera to Bartha-K61 was typically 20- to 40-fold. The capacity of neutralizing heterologous PRVs was lower, and the virus neutralization titer of antisera was 10- to 15-fold against SC strain and 10-fold against the novel HeN1 strain (Figure 5).

### Immunologic Protection in Sheep

To determine the protective effect of Bartha-K61 vaccine strain against PRV HeN1 challenge, we vaccinated sheep, challenged them with PRV strain HeN1 or SC, and then continuously observed them for 14 days. The control (unvaccinated) sheep challenged with HeN1 or SC strain all showed signs of disease and died. The vaccinated sheep challenged with SC strain did not show signs of disease; thus, the Bartha-K61 vaccine was confirmed to be of acceptable quality. However, 2 of 4 Bartha-K61–vaccinated sheep challenged with PRV HeN1 strain showed signs of disease and died; thus, the protection ratio was 50% (Table 4). These data suggest that the Bartha-K61 vaccine does not provide effective protection against infection with PRV strain HeN1.

**Table 4:** Protective effect of Bartha-K61 vaccine in sheep challenged with PRV strains HeN1 and SC, China, 2012*

Group no.	Vaccinated with Bartha-K61	No. PRV antibody–positive before challenge/no. total	Challenge virus	No. sick after challenge/no. total	No. deaths after challenge/no. total	No. protected sheep/no. total
1	Yes	4/4	HeN1	2/4	2/4	2/4
2	No	0/3	HeN1	3/3	3/3	0/3
3	Yes	3/4	SC	0/4	0/4	4/4
4	No	0/3	SC	3/3	3/3	0/3

*Sheep from a farm determined to be free from PRV infection were randomly assigned to 4 isolation rooms (Groups 1–4). After vaccination, sheep were challenged with PRV strain HeN1 or SC and continuously observed for 14 days. PRV, pseudorabies virus.

## Discussion

Pigs are the reservoir host for PRV. Nonpregnant adult pigs do not show obvious clinical signs and symptoms of infection, except for weight loss; however, among pregnant sows, the disease causes stillbirths and the birth of weak piglets with neurologic symptoms that lead to death. Newborn piglets are difficult to rear without sows, and they are not suitable for testing the pathogenicity of PRV or immunity to PRV. However, sheep of all ages are highly susceptible to PRV, even with low-dose infections, and they have high death rates from the disease. Typical symptoms observed in PRV-infected sheep are itching, constantly rising and falling head, convulsions, salivation, and difficulty with breathing; up to 100% of infected sheep die (*4*). Thus, in China, sheep are typically used for determining the efficacy of Bartha-K61 vaccine in quality standards for veterinary biologic products (*14*). In addition, lethal challenge with 1,000 LD_50_ PRV SC strain has also been documented in these quality standards.

The findings in this study showed that sheep vaccinated with Bartha-K61 vaccine were protected from a lethal challenge with PRV SC strain, proving that the vaccine was effective. However, only half of the sheep vaccinated with Bartha-K61 vaccine survived challenge with the novel HeN1 strain, suggesting that the vaccine does not provide full protection against this PRV strain. In China, PRV Bartha-K61 vaccine has been widely applied in the field for ≈30 years, and is recognized as an excellent vaccine strain. Nevertheless, since late 2011, cases of pseudorabies have occurred on many farms, and the disease has gradually become widespread in China. Animals on the affected farms had been vaccinated according to normal procedures with PRV Bartha-K61 vaccine. Brain tissue was collected from dead piglets with suspected pseudorabies on 15 pig farms in 6 China provinces and tested for the presence of PRV by PCR and virus isolation. Wild PRV was present in all 15 samples, and *gE* gene sequencing showed the isolates to be phylogenetically distant from previously characterized PRV isolates.

Newborn piglets infected with virulent PRV occasionally show diarrhea, neurologic signs, and a higher risk for death, all of which can lead to a misdiagnosis of highly pathogenic porcine reproductive and respiratory syndrome, classical swine fever, or pig epidemic diarrhea. Viremia is not evident in PRV-infected pigs (*19*), and our results showed that PRV was found at a considerably higher rate in brain tissue than in blood; thus, brain tissue is likely to be the most reliable clinical sample for diagnosing PRV infection.

PRV antibody can be detected in sheep after vaccination. However, protection against PRV challenge is not closely related to the level of antibody because the virus is nonviremic and spreads predominantly by mucosal infection and neuronal innervation (*19*). Results of a microneutralization assay suggested that serum generated by pigs vaccinated with Bartha-K61 vaccine had neutralizing ability; however, this neutralizing ability was substantially decreased for currently circulating virulent PRV strains. Moreover, there was no correlation between the neutralizing antibody titer and protection rate, so evaluation of vaccine efficacy should not be judged purely by the levels of neutralizing antibodies. Thus, the protective immune response afforded by existing vaccine strains against the currently circulating, virulent PRV HeN1 isolate remains to be elucidated.

The control and prevention of pseudorabies requires depopulation of infected animals, zoning for restricted movement of commercial animals, and improved strategies for detecting PRV infection and vaccinating against the disease (*3*). Vaccination and DIVA (differentiating infected from vaccinated animals) form the basis of control and prevention (*20*). In some European Union countries, the disease has been well controlled or eradicated by using a gE-deleted vaccine along with gE ELISA for the differential diagnosis of infection with field PRV or the vaccine strain in pigs (*21**,**22*). In China, the epidemiologic surveillance of PRV has been strengthened, PRV-positive pigs are being separated from noninfected pigs, and PRV-free pig farms have been advised to vaccinate their animals. In addition, research on the pathogenicity of PRV is ongoing in China, and a new effective vaccine is also in development in China.
